# The Post-Translational Role of UFMylation in Physiology and Disease

**DOI:** 10.3390/cells12212543

**Published:** 2023-10-29

**Authors:** Xingde Wang, Xingzhi Xu, Zhifeng Wang

**Affiliations:** Guangdong Key Laboratory for Genome Stability & Disease Prevention and Carson International Cancer Center, Marshall Laboratory of Biomedical Engineering, Shenzhen University Medical School, Shenzhen 518060, China; wangxingde2021@email.szu.edu.cn

**Keywords:** post-translational modification, UFMylation, ubiquitin-like proteins

## Abstract

Ubiquitin-fold modifier 1 (UFM1) is a newly identified ubiquitin-like protein that has been conserved during the evolution of multicellular organisms. In a similar manner to ubiquitin, UFM1 can become covalently linked to the lysine residue of a substrate via a dedicated enzymatic cascade. Although a limited number of substrates have been identified so far, UFM1 modification (UFMylation) has been demonstrated to play a vital role in a variety of cellular activities, including mammalian development, ribosome biogenesis, the DNA damage response, endoplasmic reticulum stress responses, immune responses, and tumorigenesis. In this review, we summarize what is known about the UFM1 enzymatic cascade and its biological functions, and discuss its recently identified substrates. We also explore the pathological role of UFMylation in human disease and the corresponding potential therapeutic targets and strategies.

## 1. Introduction

Post-translational modification (PTM) refers to the addition of chemical groups to one or more amino acid residues; such additions can substantially change the biological activity of the target protein [1]. To date, more than 500 different protein PTMs have been identified, including phosphorylation, glycosylation, acetylation, and ubiquitination [2]. Focusing on the latter, ubiquitin (Ub) is a small protein weighing approximately 8.5 kDa and comprising 76 amino acids. Ub is widely distributed in all eukaryotic cells and has been highly conserved during evolution. Indeed, yeast and human ubiquitin differ by only three amino acids. Ubiquitination refers to the covalent binding of Ub to target proteins, and generally requires the synergistic action of three ubiquitinating enzymes: E1 ubiquitin-activating enzyme, E2 ubiquitin-conjugating enzyme, and E3 ubiquitin-ligase [3]. First, ubiquitin is activated by E1 using energy provided by ATP hydrolysis, then transferred to E2, and, finally, covalently linked to the lysine residues of substrates with the aid of E3. Ubiquitination is a tightly regulated and reversible process: deubiquitinating enzymes (DUBs) can reverse ubiquitination by hydrolyzing the peptide or isopeptide bonds between ubiquitin molecules or between ubiquitin and substrate proteins [3]. Ubiquitination helps to regulate numerous biological processes, encompassing immune responses [4,5], the DNA damage response [6,7], cell cycle regulation [8], autophagy [9], epigenetic modulation [10], cellular apoptosis [11], and protein degradation [12], by regulating protein structures, interactions, activities, and even subcellular localizations [3].

The many ubiquitin-like proteins (UBLs) identified to date include small ubiquitin-like modifiers (SUMOs), neural precursor cell-expressed developmentally downregulated 8 (NEDD8), and interferon-stimulated gene 15 (ISG15). Although most UBLs do not necessarily share notable sequence homology with Ub, they all share a similar tertiary structure [13]. Similar to Ub, UBLs can covalently bind to target proteins (UBLylation) through a series of enzymatic reactions, similar to those involved in ubiquitination, to confer different biological functions to the substrate [14].

Ubiquitin-fold modifier 1 (UFM1) is a novel UBL formed from an 85-amino acid precursor (pro-UFM1) that is translated in human cells. UFM1 is evolutionarily conserved in multicellular organisms, but is absent in yeast [15]. Similar to Ub, the C-terminal serine and cysteine of the UFM1 precursor can be removed using specific proteases to expose the C-terminal glycine, leaving mature UFM1 to directly and covalently attach to the lysine residues of target substrates [16]. UFM1 is a unique UBL as it has only one glycine residue at its C-terminus. In addition to mono modification, UFM1 can link with other UFM1 molecules to generate UFM1 chains in a process known as poly-UFMylation [17]. Although UFM1 contains six lysines, K69 seems to be the only one that mediates poly-UFMylation. For example, it has been shown that the activating signal coactivator 1 (ASC1) is poly-UFMylated only through the K69 linkage; however, we cannot exclude the possibility that other substrates are poly-UFMylated through linkages with other lysine residues [17]. In this review, we focus on the UFMylation enzymatic cascade, the UFMylated substrates, and their roles in various cellular activities and pathological processes, hoping to shed light on potential therapeutic targets and strategies.

## 2. UFMylation

**Figure 1:**
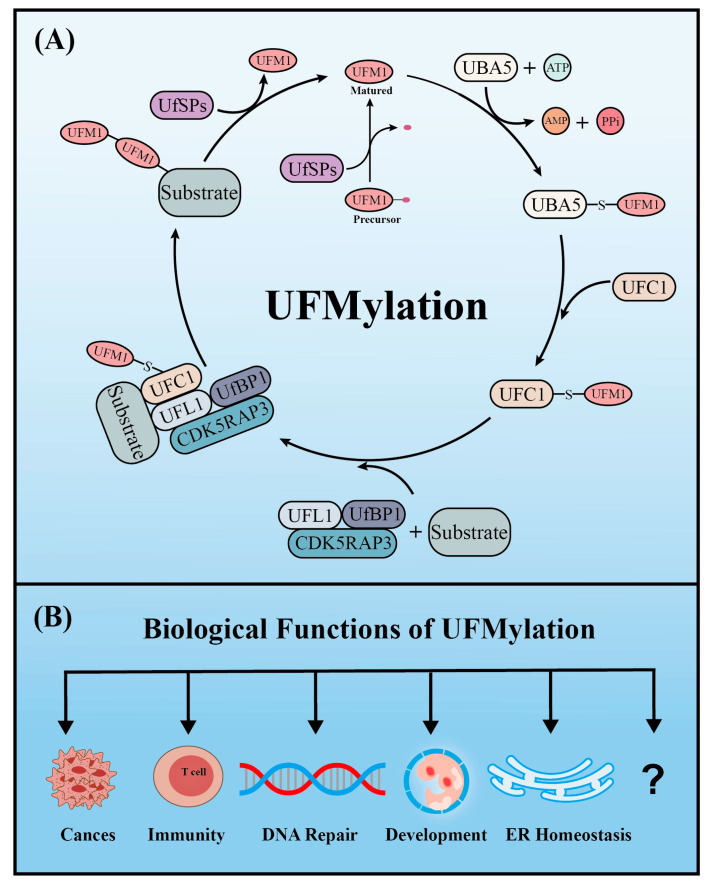
The enzyme cascade of UFMylation (**A**) and five key biological functions of UFMylation (**B**).

Similar to ubiquitination, UFM1 covalently binds to its target proteins through a cascade involving three enzymes. As described above, the *UFM1* gene is translated into a precursor form and the C-terminal glycine must be exposed by UFM1-specific proteases (UfSPs) before subsequent enzymatic reactions can occur [16]. First, when ATP is present, the E1-like enzyme UBA5 activates the mature UFM1, which forms a high-energy thioester bond between the catalytic cysteine (Cys250) of UBA5 and the exposed C-terminal glycine of UFM1. Next, the E2-like enzyme UFC1 interacts with the UFC1 binding domain of UBA5, which transfers the activated UFM1 to UFC1 by forming a similar thioester bond between UFM1 and the catalytic cysteine (Cys 116) of UFC1. Finally, UFM1 is coupled to the lysine residues of its target proteins in a process mediated by the E3-like enzyme UFL1 [18]. Another similarity with ubiquitination is that UFMylation is also reversible. In addition to maturing UFM1, UfSPs also cleave UFM1 from its target protein, thereby rendering UFM1 and its substrates recyclable (Figure 1A) [16]. Numerous verified substrates of UFM1 have been discovered and the impact of UFMylation on their functions elucidated (Table 1). 

### 2.1. UfSPs

Pro-UFM1 maturation and the removal of UFMylation from substrates are mediated by the cysteine proteases UfSP1 and UfSP2, respectively. It was, until recently, thought that UfSP2 was the only active protease because UfSP1 apparently lacks a catalytic domain [16]. However, two independent groups recently found that UfSP1 actually utilizes a non-canonical start codon (217CUG) upstream of its canonical counterpart (445AUG) to initiate translation, which produces a catalytically active UfSP1. Cong et al. reported that both UfSP1 and UfSP2 can mediate the maturation of pro-UFM1 and the de-UFMylation of substrate proteins [34]. By contrast, a study by Kulathu et al.’s group indicated that UfSP2, but not UfSP1, de-UFMylates the ribosomal subunit RPL26, while UfSP1 removes the constitutively autoinhibitory UFMylation of UFC1, thereby promoting the activation of UFMylation [20]. Interestingly, neither of the two UfSPs share significant sequence homology with known Ub-like protein-specific proteases (ULPs) or DUBs; however, they possess highly conserved cysteine and histidine residues [35,36], indicating that they might constitute a new subfamily of cysteine proteases.

### 2.2. UBA5

**Figure 2:**
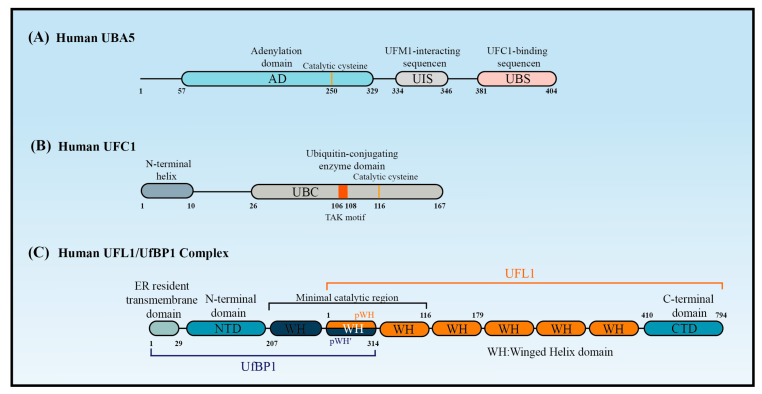
The structure of UBA5, UFC1, and UFL1/UfBP1 complex. Schematic of (**A**) the key domains of UBA5, (**B**) the key domain features of UFC1, and (**C**) the domains of the UFL1-UfBP1 E3 ligase complex.

The canonical E1 enzymes that mediate ubiquitination have conserved adenylation domains, catalytic cysteine domains, and ubiquitin-fold domains. By contrast, as a member of the non-canonical E1 enzyme family, UBA5 does not contain a catalytic cysteine domain. Instead, its catalytic cysteine active site (Cys250) is located in its adenylation domain [37] (Figure 2A). This domain comprises an eight-stranded beta sheet that is surrounded by helices [38] and it promotes UBA5 homodimer formation with pseudo-two-fold symmetry [39]. Interestingly, UBA5 dimerization is critical for UFM1 activation, but it is not stable in solution [40]. A 13-amino acid sequence called the UFM1-interacting sequence (UIS), which is located at the C-terminal of the adenylation domain, is required for UFM1’s binding activity [41,42]. This binding stabilizes the UBA5 dimer and promotes its binding to ATP [43]. Although a fragment that included the adenylation domain and UIS was proven sufficient to activate UFM1 [27,30], the short sequence at the C-terminus of UBA5 is required for UFC1 binding and the transfer of UFM1 to UFC1 [44].

Short and long UBA5 are two distinct isoforms of UBA5 encoded by the human genome, with the latter distinguished by the presence of a 56-amino acid extension at the N-terminal adjacent to the adenylation domain. Structural and biochemical studies indicated that the binding ratio of UBA5 to ATP in the presence of the N-terminus (long UBA5) is 1:1 rather than the 2:1 that occurs in its absence (short UBA5). This finding indicates that the N-terminus greatly increases the affinity of UBA5 for ATP, thereby promoting UFM1 activation at low ATP concentrations [40]. The N-terminal extension also enhances the thermal stability of UBA5 and promotes the faster transfer of UFM1 to UFC1 through a conformational change occurring at the N-terminus of UBA5 when ATP binds to the UBA5-UFM1 complex. Therefore, ATP and the binding of UFM1 to UBA5 stabilize the UBA5 homodimer, enhance UBA5 stability, and promote UFM1 transfer to UFC1.

### 2.3. UFC1

UFC1 is mainly localized in the nucleus, with a minor proportion located in the cytoplasm [15]. UFC1 lacks some features that are conserved in other E2s, such as the catalytic histidine–proline–asparagine (HPN) motif, which suggests it has a unique mechanism of function and modulation [45]. Some ubiquitin E2 enzymes bind to Ub to promote E2 dimerization, stabilize E2s in their active-closed state, and enhance their catalytic efficiency [3]. It is unknown whether UFC1 activity is regulated by similar mechanisms.

Although UFC1 differs from the other E2 enzymes, it contains a catalytic core structural domain that is conserved in all E2 enzymes. This catalytic core domain presents as a flexible loop formed of approximately 10 amino acid residues and confers strong solvent accessibility [46]. The loop encloses the intermediate active cysteine residue (Cys116), which undergoes a trans-esterification reaction upon the transfer of UFM1 from UBA5 to UFC1 [46] (Figure 2B). Interestingly, the highly conserved HPN motif contained in all E2 enzymes is replaced by a threonine–alanine–lysine (TAK) motif in UFC1. Mutations within the TAK motif (T106I and K108A) impair UFC1 function and are associated with human diseases, such as encephalopathy [45]. In addition to the catalytic core structural domain, UFC1 contains an N-terminal helix that is not present in other E2s. Structural studies have indicated that this helix can adopt various conformations to suit different substrates. Moreover, the N-terminal helix-truncated form of UFC1 (UFC1^ΔN^) has stronger UFMylating activity compared with the wild-type UFC1 (UFC1^WT^) in vitro, indicating that the N-terminal helix has an inhibitory role in the UFMylation enzyme cascade. Although further confirmation is required, it seems that UFC is auto-UFMylated on K122, the residue downstream of the catalytic C116. This event leads to the inhibition of the UFC1 activity that is dependent on its N-terminal helix [20]. Furthermore, CDK5 regulatory subunit-associated protein 3 (CDK5RAP3) has no inhibitory effect on the UFMylating activity of UFC1^ΔN^ in vitro, confirming that the N-terminal helix of UFC1 mediates the ability of CDK5RAP3 to inhibit UFMylation [47].

UFC1 may transfer UFM1 to substrates in association with UFL1/UfBP1. Data from in vitro assays confirmed that UFC1 can transfer UFM1 to free cysteines but not free lysines, indicating that UFC1 cannot transfer UFM1 to substrates directly. UFC1 together with UFL1/UfBP1 can, however, transfer UFM1 to free lysines [47]. Strangely, none of the cysteine mutations so far applied to UFL1 have influenced its activity, and no transthiolation products of UFL1/UfBP1 have been observed [47]. Therefore, given that UfBP1 does not have any cysteine residues, UFC1 may transfer UFM1 to substrates with the aid of UFL1/UfBP1 as scaffolding proteins.

### 2.4. UFL1 and UfBP1

UFL1 contains a transmembrane domain enabling its primary localization on the cytoplasmic side of the endoplasmic reticulum (ER) membrane. UFL1 can, however, also be found in the cytoplasm and nucleus due to the presence of a nuclear localization signal [48]. In the UFMylation system, UFL1 is the only E3 ligase identified so far that aids the transfer of UFM1 to target substrates [49]. Notably, UFL1 does not contain RING, HECT, or RBR domains, implying that UFL1 lacks a catalytic cysteine site that can accept UFM1 from UFC1 [49].

Hundreds of E3 ligases that participate in ubiquitination are classified into three types according to domain structures. RING E3s contain the very interesting new gene domain, while HECT E3s contain a domain that is homologous to the E6-AP C-terminus domain, and RBR E3s contain a RING-between-RING domain. RING E3s transfer ubiquitin directly from the E2s to the substrates without binding to the Ub, while HECT and RBR E3s require Ub to first form a thioester bond with the conserved cysteine before the Ub is transferred to the substrates [50]. Notably, UFL1 contains neither a RING domain, nor the conserved cysteine-containing HECT and RBR domains [49], implying that UFL1 is a non-canonical E3 ligase. This fact raises the question as to the role of UFL1 in transferring UFM1 from UFC1 to substrates. Data from in vitro UFMylation assays show that the UFMylation of UFL1 and its substrates were not affected by any of the single mutations of Cys to Ala in UFL1, indicating that UFL1 lacks a catalytic Cys that can accept UFM1 from UFC1. Therefore, similar to the RING E3s, but not the HECT or RBR E3s, UFL1 functions as a scaffolding protein that brings UFC1 and its substrates together [47].

Interestingly, UFL1 cannot function properly alone, but requires UfBP1 to promote its stability and activity. UfBP1, also known as C20orf116 or DDRGK1, is a UFM1-interacting protein composed of 314 amino acids that include a C-terminal proteasome-COP9-initiation factor domain. UfBP1 also contains a transmembrane domain and is localized on the cytoplasmic side of the ER membrane, where it exists in a complex with UFL1 [19]. Intriguingly, the loss of UfBP1 affects the stability and expression levels of *UFL1* [19,49,51,52]. *UFL1* expressed alone in *E. coli* forms inclusion bodies, while UfBP1 inhibits the inclusion body formation, suggesting that UfBP1 helps UFL1 retain a stable and functional state [47]. More importantly, UFL1 functions as an E3 ligase only when it forms a complex with UfBP1. Indeed, studies on the UFMylation of ASC1 and RPL26 showed that UfBP1 greatly enhances UFL1 E3 ligase activity [47]. An in vitro assay showed that neither UFL1 nor UfBP1 have the active cysteine residues required to accept UFM1, and that UFC1 transfers UFM1 to substrates only in the presence of the UFL1/UfBP1 complex [47], suggesting that UFL1/UfBP1 functions as a scaffold-type E3 ligase that binds charged UFC1 to promote aminolysis.

Structure predictions have shown that UFL1 and UfBP1 form a heterodimer composed of several winged helix (WH) domain repeats [47] (Figure 2C). UFL1 has an N-terminal helix followed by a partial WH (pWH) and five WH domains, which are important for its E3 ligase activity. UfBP1 has an N-terminal transmembrane segment, a long helical region followed by a WH (WH1′) domain, and a partial WH (pWH′) domain [33]. The partial pWH domain at the N-terminus of UFL1 complements the partial pWH′ domain at the C-terminus of UfBP1 to form a composite WH (pWH-pWH′) domain that is essential for complex formation and protein stability [33]. Data from in vitro assays showed that UFL1 (pWH-WH1) and UfBP1 (WH1′-pWH′) together are sufficient for the transfer of UFM1 from UFC1 to target substrates [47].

CDK5RAP3 is another protein that is consistently associated with UFL1. Because CDK5RAP3 always functions as a substrate adaptor [53], it is thought that CDK5RAP3, together with UFL1/UfBP1, forms part of an integral E3 ligase complex [51,54,55]. CDK5RAP3 binds to the ligase complex of UFL1/UfBP1 and restricts its E3 ligase activity. CDK5RAP3 functions as a specificity determinant, inhibiting ligase activity in the absence of a substrate and directing ligase activity toward the ribosomal subunit RPL26 [47].

## 3. Biological Functions of UFMylation

Like ubiquitination, UFMylation has important functions in multiple biological processes and diseases. To date, UFM1 modification has been reported to be involved in processes including the DNA damage response, ER stress, hematopoiesis, fatty acid metabolism, and G-protein coupled receptor (GPCR) biogenesis. Furthermore, aberrant UFM1 cascades are reportedly associated with several human diseases, such as cancer, ischemic heart disease, diabetes, atherosclerosis, hip dysplasia, and schizophrenia. In this section, we summarize the functions of UFMylation in the DNA damage response, ER stress, ER-phagy, autophagy, immune responses, developmental diseases, and cancers (Figure 1B).

### 3.1. UFMylation and the DNA Damage Response

DNA damage caused by endogenous or exogenous factors seriously impairs genomic integrity but can be rescued via DNA damage response pathways [56]. DNA double-strand breaks (DSBs), which are extremely toxic to cells, are repaired almost exclusively by homologous recombination (HR) and non-homologous end-joining. Emerging evidence indicates that UFMylation has numerous important roles in mediating the cellular response to DSBs, thus contributing to the maintenance of genome stability and preventing tumorigenesis [57].

#### 3.1.1. MER11 UFMylation

UFL1 co-localizes with γH2AX, a marker of DSBs, during UV- and IR-induced DNA damage. In addition, UFM1 and UFL1 are immediately recruited to laser-induced DSBs [25,26]. These observations have prompted us to speculate that UFMylation has a role in the DSB response. At the initiation of the DSB response, the MRE11-RAD50-NBS1 (MRN) complex re-localizes to the damage sites in what is regarded as the first and most important step that activates the kinase of ataxia telangiectasia mutated (ATM) [56]. UFL1 depletion inhibited the activation of ATM after DSB formation [57], indicating that UFMylation plays important roles in ATM activation. The interaction between UFL1 and the MRN complex, together with the decreased recruitment and stability of the MRN complex resulting from UFL1 depletion, led to the identification of MRE11 UFMylation on K282 [25,57]. MRE11 is a core factor in the MRN complex and binds directly to RAD50 and NBS1. This complex is integrally recruited to damage sites, which promotes ATM activation and DNA end resection, thereby promoting HR repair [56]. We showed that MRE11 UFMylation deficiency inhibits MRN complex formation, its recruitment to damage sites, and, subsequently, DSB-induced ATM activation [25]. The UFMylation of MRE11 also promotes HR-mediated DSB repair and genome stability [25]. Interestingly, a pathogenic mutation of MRE11 (G285C) surrounding K282 identified in in uterine endometrioid carcinoma after searching The Cancer Genome Atlas (TCGA) was found to compromise MRE11 UFMylation [25,57]. Further studies showed that MRE11 G285C mutation is associated with cellular phenotypes that are similar to that of the UFMylation-deficient mutant MRE11 (K282R) [25,57], suggesting that MRE11 UFMylation is closely associated with tumorigenesis.

The function of MRE11 UFMylation in the DSB response is also mediated by UfSP2 [58]. Evidence from human cell lines showed that UfSP2 is phosphorylated at serine 374/381 by ATM, which promotes its release from the MRN complex after the formation of DSBs [25,58]. This release enhances MRE11 UFMylation and ATM activation, which, in turn, further promotes UfSP2 phosphorylation. UfSP2 is dephosphorylated by the phosphatase WIP1, which promotes UfSP2 recruitment to DSBs, leading to the de-UFMylation of MRE11 and H4, and suppresses ATM activation [58].

Besides its functions in relation to DSBs, the UFMylation of MRE11 is essential for telomere length maintenance and hematopoietic stem cell survival [59]. UFL1- and UFM1-deficient zebrafish exhibited telomere shortening associated with developmental delay, impaired hematopoiesis, and premature aging compared to wild-type zebrafish [59]. Furthermore, MRE11 UFMylation promoted the dephosphorylation of NBS1 by PP1α to facilitate NBS1 interactions with TRF2, thus preventing premature senescence and telomere shortening in zebrafish [25].

#### 3.1.2. Histone H4 UFMylation

Another mechanism by which UFMylation regulates DSB-induced ATM activation involves UFL1 phosphorylation at serine 462 by ATM. This event promotes UFL1 recruitment to sites of DNA damage. UFL1 catalyzes the mono-UFMylation of histone H4 on K31 [26], which can be recognized by the UFMylation reader serine/threonine kinase 38 (STK38) [60]. STK38 contains a UFM1-binding motif that when mutated at conserved amino acids can no longer interact with UFMylated H4. STK38 recruitment to DNA damage sites also depends on the monoacylation of H4 and is critical for the subsequent recruitment of SUV39H1. SUV39H1 catalyzes the trimethylation of H3K9 (H3K9me3), which binds to and recruits Tip60. In turn, Tip60 acetylates ATM and promotes its activation [26]. The UFMylation of H4 is also regulated by UfSP2 and its ATM-induced phosphorylation (see MRE11 above).

MRE11 UFMylation at DSBs is first catalyzed by UFL1 to promote MRN complex recruitment and ATM activation. Active ATM then phosphorylates UFL1 and UfSP2. The phosphorylation of UFL1 enhances its recruitment to sites of DNA damage, while the phosphorylation of UfSP2 releases it from the MRN complex, and both processes further increase MRE11 UFMylation. UFL1 and UfSP2 phosphorylation also leads to enhanced H4 UFMylation and, later, ATM activation. The phosphatase WIP1 de-phosphorylates UfSP2, which de-UFMylates MRE11 and H4, and finally reverses ATM activation.

**Figure 3:**
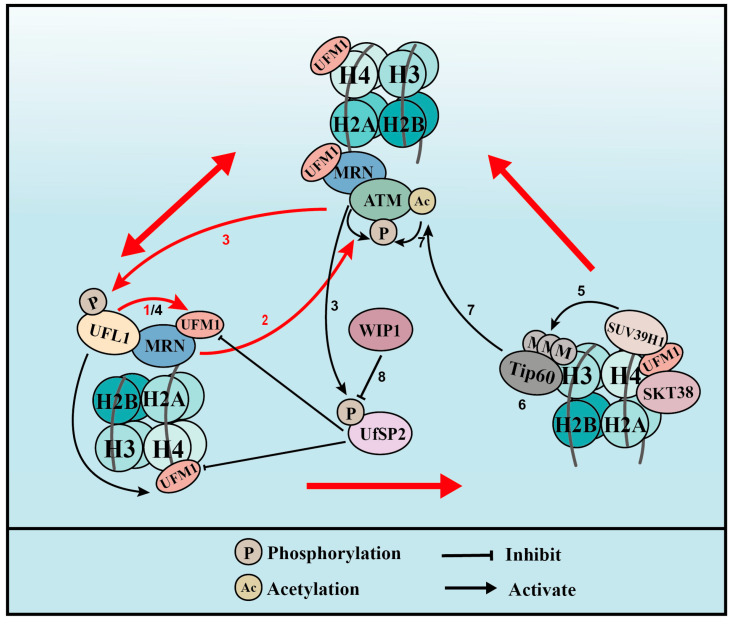
UFMylation of MRE11 and H4 promotes ATM activation (for the purposes of the diagram, MRE11 is abbreviated to MRN complex). (**1**) Upon DNA damage, UFL1 is recruited to chromatin via MRE11 to UFMylate MRE11, which promotes the formation and recruitment of the MRE11-RAD50-NBS1 (MRN) complex. (**2**) UFMylated MRE11, together with RAD50 and NBS1, activates ATM. (**3**) Activated ATM further phosphorylates UFL1 and UfSP2, which promotes UFL1 recruitment, while inhibiting UfSP2 recruitment. (**4**) As a positive feedback loop, phosphorylated UFL1 further enhances MRE11 UFMylation and UFMylates H4. (**5**) UFMylated H4 can be recognized by the UFMylation reader serine/threonine kinase 38 (STK38), which is critical for the subsequent recruitment of the methyltransferase SUV39H1. (**6**) SUV39H1 trimethylates lysine 9 of histone H3 (H3K9me3), which recruits Tip60. (**7**) Tip60 acetylates ataxia telangiectasia mutated (ATM) and promotes ATM activation. (**8**) Lastly, the phosphatase WIP1 de-phosphorylates UfSP2 and reverses ATM activation. Red arrows indicate the two positive feedback loops.

UFMylation therefore promotes ATM activation though two distinct positive feedback loops: one is WIP1-UFL1/UfSP2-MRE11-ATM-UFL1/UfSP2 phosphorylation and the other is WIP1-UFL1/UfSP2-H4-STK38-SUV39H1-H3K9me3-Tip60-ATM acetylation-ATM phosphorylation-UFL1/UfSP2 phosphorylation (Figure 3).

### 3.2. UFMylation in ER Metabolism

#### 3.2.1. ER Stress

ER stress is caused by an overload of un/mis-folded proteins in the ER. In mammalian cells, three ER transmembrane proteins act as sensors of ER stress: activating transcription factor 6, inositol-required enzyme 1α (IRE1α), and PKR-like ER kinase (PERK) [61]. The accumulation of un/mis-folded proteins triggers ER stress and activates un/mis-folded-protein response (UPR) signaling to the ER to enhance its protein processing capacity. Therefore, the amount of UPR signaling is representative of ER stress levels. The un/mis-folded-proteins are then degraded through a type of ubiquitin-proteasome degradation known as ER-associated degradation (ERAD) [61]. Damaged ER can also be removed through an autophagy process called ER-phagy [62]. Thus, ERAD and ER-phagy are the predominant pathways involved in alleviating ER stress.

As mentioned above, UFL1 and UfBP1 are localized on the ER membrane, which suggests that UFMylation is involved in ER stress. The first evidence that UFMylation is related to ER stress was obtained in studies to determine the involvement of ER stress in the development of heart disease [63]. Later, numerous studies showed that the UFMylation substrate UfBP1, an UFL1 partner, could respond to ER stress [17,49,64], and CDK5RAP3 could sense proteotoxic stress in the ER lumen by forming a tripartite receptor complex with the ER-associated UFL1/UfBP1 [65]. In another example, cisplatin induced a stress-related increase in UFL1 expression in granulosa cells and enhanced ER stress, processes which were exacerbated by UFL1 knockdown and alleviated by UFL1 overexpression [66]. Others have reported that UFMylation modulates UPR signaling. For instance, UFL1 depletion generally increased the cellular levels of phosphorylated eIF2α (p-S51-eIF2α) and spliced XBP1 (XBP1s) mRNA [67], which reflect ER stress levels. The depletion of UfBP1 and UBA5 also significantly upregulated the cellular levels of p-S51-eIF2α and XBP1s mRNA [19,68]. UfBP1 regulates IREα protein stability during their interaction, a process that is dependent on its UFMylation [68]. Moreover, the IREα/XBP1 axis upregulates the expression of UfBP1 and UFMylation pathway genes in plasma cells, while UfBP1 deficiency promotes the activation of PERK, which impairs ER expansion in plasma cells and retards immunoglobulin production. Results from structure and function analyses suggest that K267 in UfBP1, the main lysine that undergoes UFMylation, is not required for the development of plasmablasts, but it is required for immunoglobulin production and stimulating ER expansion in IRE1α-deficient plasmablasts [52]. Together, this evidence reveals a regulatory role of UFMylation in ER stress.

CDK5RAP3, another UFL1 partner, is also involved in ER stress, especially the expression of XBP1 and PERK [69]. During the interphase of the cell cycle, microtubules are predominantly nucleated at the centrosome (microtubule organizing centers; MTOC) by γ-tubulin ring complex (γTuRC) proteins. Most γTuRCs are activated by structural rearrangement, phosphorylation, or binding to modulating proteins accumulated in MTOCs [69]. The interaction of ER membranes with newly formed microtubules could promote ER expansion and help to restore ER homeostasis. UFL1 can interact with CDK5RAP3 and form a complex with γTuRCs [70,71], negatively regulating microtubule nucleation at interphase centrosomes. In mammalian cells, ER network rearrangements largely depend on interactions with dynamic microtubules. Therefore, an UFL1/CDK5RAP3 deletion induces ER stress and the release of γTuRC proteins, which in turn stimulates microtubule nucleation. Thus, the interaction between the ER and newly formed microtubules promotes ER enlargement to restore ER homeostasis. Prolyl 4-hydroxylase beta (P4HB), another UFMl substrate, is also involved in ER stress. P4HB has oxidoreductase, chaperone, and isomerase functions, which prevent protein misfolding and ER stress [72,73,74], and help to regulate reactive oxygen species (ROS) production and mitochondrial function [75,76]. P4HB UFMylation at K69/114/130 regulates its stability, while defective P4HB protein UFMylation promotes its degradation via the ubiquitin-proteasome pathway. This degradation event causes mitochondrial function damage as well as oxidative and ER stress [33].

#### 3.2.2. ER-Phagy

UFMylation has an important role in ER-phagy. Data from several recent studies have confirmed that UFMylation regulates ER degradation through lysosomes, thus furthering our understanding of the mechanisms by which UFMylation regulates ER stress. A human genome-wide screen indicated that UfBP1 is an ER-phagy regulator [22]. Many factors associated with the ribosome and translational quality control, such as RPL26 and RPN1, were identified as UFMylation substrates that mediate ER-associated autophagy [22,24,77]. NADH-cytochrome b5 reductase 3 (CYB5R3) is also UFMylated, and this mediates its degradation by lysosomes [23]. In addition, CDK5RAP3 was found to bind to the autophagosome-localizing autophagy-related protein 8 family of proteins [65].

CDK5RAP3 has been proposed to function as both a substrate adaptor that directs UFMylation toward target substrates, such as ribosomal protein RPL26 [47], and an adaptor protein for UFMylation-dependent ER-phagy [23,65]. The results from one study showed that CDK5RAP3 depletion increased the amounts of GFP-UFM1-conjugated CYB5R3 and UfBP1, while treatment with bafilomycin A1 to suppress autophagy had no effect [23]. These results support the idea that CDK5RAP3 promotes UFMylation-mediated ER-phagy. In a second study, the CDK5RAP3/UFL1/UfBP1 complex on the ER was activated by stalled ribosomes, which induced the degradation of internal or passenger proteins in the ER [65]. Therefore, the CDK5RAP3/UFL1/UfBP1 complex and UFMylation protein mutants are highly susceptible to ER stress. Hence, CDK5RAP3 forms a ribosome-associated translation quality control pathway that bridges autophagy and ER stress.

The UFMylation of ribosomal proteins is believed to control the ribosomal stress response and ribosome-mediated protein translation quality. Indeed, UFL1 interacts with ribosomes, and the UFMylation substrate screening of ribosomal proteins showed that many ribosomal subunits are UFMylated [78]. Similarly, UfBP1-dependent UFMylation substrate screening with nutrient starvation led to the identification of several ribosomal subunits, ribosome-associated factors, and ER-resident translocon proteins as UFMylation substrates, including RPL7A, RPLP0, RPL10A, RPL30, RPL19, and RPN1 [22]. These findings suggest that ribosomal proteins are likely modified by UFMylation, although most candidates remain to be confirmed experimentally. Nevertheless, all these candidates are associated with protein translation and quality control on the ER membrane, with disruption leading to ER metabolic disturbance, indicating that their UFMylation by UFL1/UfBP1 on the ER facilitates ER-phagy and decreases ER stress.

RPL26 is UFMylated at K132 and K134 [24,77] when it is located at the ER surface, as the UFL1/CDK5RAP3/UfBP1 E3 complex is restricted to the ER membrane [24]. This modification can be upregulated after treatment with the protein translation inhibitor anisomycin, indicating that ribosome arrest during cotranslational translocation in the ER is a specific trigger for RPL26 UFMylation [77]. In addition, RPL26 UFMylation promotes translocation-arrested ER protein degradation by lysosomes [24]. While UFMylation deficiency leads to increased ER stress, impaired ERAD, and ER-phagy [22,24], RPL26 UFMylation induces ER fusion with lysosomes and presents stalled nascent proteins for degradation, thereby serving as a form of protein translation quality control in the ER. Another ribosomal protein RPL10, a regulator of actively translating ribosome formation [79], is reportedly UFMylated to significantly increase cell proliferation in pancreatic adenocarcinoma (PAAD) [28]. Whether RPL10 also regulates ER-phagy is a subject for further research.

CYB5R3 is another master regulator of ER-phagy and is UFMylated at K214 [23] while it is anchored to the ER membrane. The researchers who used genome-wide CRISPR screening to identify UFL1 and UfBP1 as activators of ER-phagy [22] explored whether CYB5R3 UFMylation is involved in this process. They showed that CYB5R3 contains FAD- and NADH-binding domains and catalyzes the transfer of reducing equivalents from NADH to cytochrome b5, which then acts as an electron donor [80]. Interestingly, K214 of CYB5R3, the conjugation site for UFM1, is at the interface between the NADH- and FAD-binding domains, so the modification of the conjugation site disrupts the conformation equilibrium and reduces its enzymatic activity. An ER-phagy reporter was used to show that UFM1-modified CYB5R3 is degraded by lysosomes [23]. The ER is delivered to lysosomes via two main mechanisms: macro-ER-phagy, which is associated with autophagosome formation around the targeted ER subdomain, and micro-ER-phagy, wherein lysosomes invaginate and surround the targeted ER subdomain [81,82]. After nutritional starvation-induced ER-phagy, UFMylated CYB5R3 colocalized with core autophagy-related gene (ATG) machineries, such as FIP200, WIPI2, and LC3, which are essential for autophagosome formation [23]. These data suggest that CYB5R3 UFMylation functions as a signal for macro-ER-phagy.

#### 3.2.3. Autophagy

Although UFMylation regulates ER-phagy, the involvement of this PTM in general autophagy remains uncertain. Results from one study indicated that UFL1 deficiency impairs autophagy activity. LC3B associates with autophagosome development and maturation [83] and p62/SQSTM1 serves as a bridge between LC3 and polyubiquitinated proteins, which are selectively packaged into autophagosomes. Therefore, LC3B and p62/SQSTM1 reflect the levels of autophagy [84]. Indeed, UFL1 depletion in bone marrow (BM) cells resulted in increased ER stress and an increase in the abundance of LC3B and p62/SQSTM1, indicating that UFMylation regulates ER stress and general autophagy [67]. However, the knockout of UfBP1 in BM cells did not influence the levels of LC3B or p62/SQSTM1 [19]. As UfBP1 normally functions synergistically with UFL1 to promote UFMylation, the opposite effects caused by defects in these two proteins seem contradictory. Interestingly, a genome-wide CRISPR screen of neuroglioma H4 cells identified several novel modulators of p62/SQSTM1, including the UFMylation cascade, which regulates p62/SQSTM1 expression by eliciting a cell-type-specific ER stress response, although few LC3B expression changes were evident when UFM1 was depleted [85]. Because the depletion of three UFMylation-related proteins resulted in three different results, the role of UFMylation in autophagy remains controversial. Although UfBP1 and RPL26 UFMylation control ER protein homeostasis and ER-phagy [22,77], no alterations inLC3B and p62/SQSTM1 were detected when UfBP1 was depleted [22]. Considering these discrepancies, more research is needed to conclusively determine whether UFMylation modulates general autophagy.

### 3.3. UFMylation and Development

UFMylation has an important impact on embryonic development. The complete depletion of UBA5 is embryonically lethal, with most UBA5^−/−^ mice embryos succumbing between 12.5 and 13.5 embryonic days (E12.5-E13.5) after gestation. By contrast, UBA5-heterozygous (UBA5^+/−^) mice are born healthy and fertile without the emergence of any noticeable pathology for at least 2 years [86]. Similarly, the complete depletion of UFL1 is embryonically lethal, with most UFL1^−/−^ mice embryos succumbing before E11.5, and as early as E10.5; UFL1^+/−^ mice are born healthy [67]. UfBP1 depletion also causes death during embryonic development. While UfBP1^+/−^ mice are born healthy, most UfBP1^−/−^ mice embryos succumb by E12.5 [19]. Finally, CDK5RAP3 depletion also results in embryonic lethality by E8.5 [87]. It thus seems that UFMylation has important roles in ontogenesis and in the development of organs and tissues, such as the erythroid and skeletal system, and the brain.

#### 3.3.1. Erythroid Development

An UFMylation deficiency causes the failed development of erythroid lineages [19,67,86]. An analysis of *UBA5*^−/−^ mouse embryos at different developmental stages revealed a marked fetal anemia phenotype compared with *UBA5*^+/+^ mouse embryos that was rescued by transgenic expression of UBA5 in the erythroid lineage [86]. The loss of UFL1 blocked autophagic degradation and increased mitochondrial mass and ROS production in bone marrow cells, leading to the DNA damage response, p53 activation, and ER stress. This ER stress and the resulting generation of UPR enhanced hematopoietic stem cell death and impaired hematopoietic development, resulting in severe anemia, cytopenia, and ultimately animal death [66]. Similarly, primitive erythropoiesis was also impaired in UfBP1-deficient embryos, and UfBP1-deficient mice exhibited severe pancytopenia [19]. In addition, increased RPL26 UFMylation was detected in an in vitro erythroid differentiation model created by treating K562 cells with hemin [77,88]. Ribosome UFMylation was also upregulated during erythropoietin-induced erythroid differentiation in primary CD34^+^ hematopoietic stem and progenitor cells [77]. Collectively, these studies have provided abundant evidence that UFMylation is indispensable for hematopoiesis and erythroid development.

#### 3.3.2. Skeletal Development

Several studies have indicated the importance of UfBP1 in cartilage growth and development. Alongside this, reports that UfBP1 mutations are involved in spondylo-epi-metaphyseal dysplasia Shohat type (SEMDSH) disease indicate that UfBP1 has important roles in skeletal development. The whole-exome sequencing of four SEMDSH-prone families revealed a splice variant of UfBP1 (c.408 + 1G > A) resulting in a premature stop codon that causes a loss of function [89]. Two unrelated SEMDSH patients were found to carry a different mutation of UfBP1 (G135K), which was associated with a similar phenotype to the UfBP1 (c.408 + 1G > A) mutation. These findings indicate that UfBP1 is associated with SEMDSH and skeletal development [90]. In support of this association, UfBP1 depletion in zebrafish embryos resulted in craniofacial defects, and the deletion of UfBP1 in mouse embryos significantly increased limb bud apoptosis and cell death. Mechanistically, UfBP1 binds directly to SOX9, a major transcription factor for chondroblasts, to inhibit SOX9 ubiquitination and proteasomal degradation. COL2A1, the downstream target of SOX9, is linked to skeletal disorders. Therefore, UfBP1 defects lead to skeletal dysplasia by disturbing the SOX9-COL2A1 axis [89]. In addition, transgenic mice with conditionally UfBP1-depleted limb mesenchymal cells exhibited limb shortening and joint abnormalities, indicating that UfBP1 helps to mediate normal cartilage growth and development [91].

#### 3.3.3. Brain Development

Many studies have implicated UFMylation in brain development. Genetic studies have revealed that variants of the human *UBA5*, *UFC1*, and *UFM1* genes are associated with a number of neurodevelopmental diseases, including infantile-onset encephalopathy [92], autosomal recessive cerebellar ataxia [93], and microcephaly [45]. Using exome sequencing, two groups found two biallelic mutations in *UBA5* (A371T and a loss-of-function nonsense mutation) that led to postnatal microcephaly, epilepsy, and spasticity in severe epileptic syndrome patients [92,93,94]. CNS-specific knockout of UFM1 in mice caused neonatal death accompanied by microcephaly and the apoptosis of specific neurons [92]. Moreover, knockout of *UBA5* and other genes of the UFM1-cascade in *Caenorhabditis elegans* resulted in altered neurotransmission. Meanwhile, *UBA5* silencing in zebrafish decreased their motility while inducing abnormal movements suggestive of seizures [94]. In addition, two biallelic *UFC1* mutations (T106I and R23Q) and one biallelic *UFM1* mutation (R81C), which impaired UFM1-UFC1 intermediate formation and resulted in a widespread reduction in cellular UFMylation, were identified in patients with severe early-onset encephalopathy [45]. An in vitro UFMylation assay using purified proteins showed that a T106I mutant of UFC1 dramatically impaired UFMylation [47]. Moreover, compared to the wild-type, CYB5R3 UFMylation-defective knock-in mice exhibited severe microcephaly [23]. 

#### 3.3.4. Development of Other Organs and Tissues

UFMylation is also involved in the development of other organs and tissues. Nephron-tubule-specific UFL1-KO mice presented kidney atrophy and interstitial fibrosis, demonstrating the crucial role of UFL1 in regulating kidney function [95]. Hepatocyte-specific UFL1-KO induced hepatocyte apoptosis and mild steatosis in mice at 2 months of age and hepatocellular ballooning, extensive fibrosis, and steatohepatitis at 6–8 months of age [96]. The deletion of *UFL1* in cardiomyocytes and intestinal epithelial cells caused heart failure and an increased susceptibility to experimentally-induced colitis, respectively, suggesting that UFL1 has an essential role in the maintenance of homeostasis in these organs [97,98]. Furthermore, intestinal epithelial cell-specific CDK5RAP3-KO mice showed an almost complete absence of Paneth cells and an increased susceptibility to experimentally-induced colitis, suggesting a key role for CDK5RAP3 in Paneth cell development and maintenance [87]. UFL1-deficient mice showed constitutive amylase secretion and the disruption of ER homeostasis [99]. Thus, evidence supports an important role for UFMylation in the development and function of various organs and tissues.

### 3.4. UFMylation and Immune Response

Recent work has shed light on the important role of UFL1 in antiviral innate immunity after DNA virus infection [100]. UFL1 protein levels were significantly downregulated when peritoneal macrophages were infected with DNA viruses, such as the herpes simplex virus (HSV-1) or vaccinia virus (VACV), which also significantly decreased the mRNA expression of interferon β1, interleukin-6, and tumor necrosis factor. These results suggest that UFL1 promotes antiviral innate immunity. Further studies showed that UFL1 regulates the cGAS-STING pathway through its effects on STING stability. The E3 ligase TRIM29 ubiquitinates STING at K338/347/370, promoting its proteasome-dependent degradation [101,102]. UFL1 competitively binds to STING to inhibit K48-linked ubiquitination, thereby maintaining STING protein stability and ultimately promoting antiviral innate immunity [100]. Notably, UFL1^ΔN^, which lacks the N-terminal domain and thus E3 ligase activity, was less effective in promoting IFN-β activation compared with the full-length UFL1, suggesting that ULF1’s E3 ligase activity is not necessary for its antiviral innate immune response. In addition, *UFC1* knockdown had no influence on the expression of interferon β1 and interleukin-6 mRNA induced by HSV-1. This evidence suggests that UFL1 regulates the antiviral innate immune response via a mechanism that is independent of UFMylation.

UFL1 also plays an important role in antiviral innate immunity after RNA virus infection. A proteomic analysis of mitochondrial-associated ER membranes during RNA virus infection revealed that UFL1 was dynamically recruited to mitochondria anti-viral signaling protein (MAVS) at ER–mitochondrial contact sites [103]. MAVS interacts with RIG-1, an important RNA-virus sensor, and further research has confirmed that UFMylation machinery proteins, including UFL1, positively regulate RIG-1 signaling and the gene transcription that follows infection with RNA viruses like Sendai virus [30]. Mechanistically, after RNA virus infection, UFL1 re-localizes to intracellular membranes and interacts with 14-3-3ε and RIG-1, promoting 14-3-3ε UFMylation. The UFMylation of 14-3-3ε induces its interaction with RIG-1 and activates MAVS, which contributes to downstream signal transduction and eventually leads to IFN production. In addition, the UFMylation of RPL26 mediated by the UFMylation machinery was also reported to be required for the optimal translation of hepatitis A virus (HAV) RNA and HAV replication [104].

### 3.5. UFMylation and Cancers

A comprehensive analysis of genomic alterations in the eight UFMylation family genes (*UFM1*, *UBA5*, *UFC1*, *UFL1*, *UfBP1*, *CDK5RAP3*, *UfSP1*, and *UfSP2*) across the TCGA database of 33 cancer types identified 55 recurrent and focal somatic copy number alteration events in UFMylation family genes [105]. Among the UFMylation genes, *UfSP2* was frequently deleted in 14 cancer types. Calculations of the frequencies of copy number gain or loss for UFMylation genes in each cancer type revealed that *UfSP2* (31%), *UFM1* (31%), and *UFL1* (28%) had the highest average frequency of copy number loss, whereas *UFC1* (34%), *UfSP1* (34%), and *UfBP1* (30%) had the highest average frequency of copy number gain [105]. In total, 11.08% of the TCGA samples had high-level copy number alterations in at least one of the eight genes [105]. Therefore, improving our understanding of the role of UFMylation in cancers might open up new avenues for therapeutic development. Since the first report that ASC1 UFMylation promotes breast cancer progression was published in 2014 [17], a huge number of studies have uncovered the significant roles of UFMylation in multiple cancers.

In a study of colon cancer, depleting UfSP2 significantly promoted the growth of tumor cells [105], while UBA5 can be upregulated in breast cancer and associated with poor prognosis [106]. The level of RPL10 UFMylation was two- to three-fold higher in PAAD tumors than adjacent normal tissues, and UFL1 KO or UfSP2 overexpression resulted in decreased rates of cell proliferation in PANC-1 and Mia PaCa-2 cells, indicating that RPL10 UFMylation can enhance cell stemness for PAAD development [28].

The long non-coding RNA B3GALT5-AS1 suppresses hepatocellular carcinoma (HCC) cell proliferation, invasion, and metastasis by regulating miR-934 and UFM1 [107]. MicroRNA miR-934 targets UFM1 to inhibit its UFM1. B3GALT5-AS1 negatively regulates miR-934, leading to high UFM1 expression, and miR-934 mimics or UFM1 downregulation reverse the inhibitory effect of B3GALT5-AS1 in HCCLM3 [107]. Furthermore, more than 50% of mice with hepatocyte-specific depletion of UFL1 and UfBP1 developed spontaneous HCC by 14 months of age. Mechanistically, this was caused by the direct interaction of the UFL1/UfBP1 complex with the mTOR/GβL complex and attenuation of mTORC1 activity. The ablation of UFL1 or UfBP1 in hepatocytes results in the dissociation of the protein from the mTOR/GβL complex and the activation of oncogenic mTOR signaling to drive HCC development [96]. These studies provide evidence of the functions of UFMylation in HCC tumorigenesis.

UFM1, UfBP1, and CDK5RAP3 expression is also downregulated in gastric cancer compared with respective adjacent non-tumor tissues; this downregulation is a poor prognostic factor for affected patients [108,109,110]. CDK5RAP3 suppresses gastric cancer development by inhibiting AKT/GSK-3β phosphorylation and negatively regulating Wnt/β-catenin signaling [111,112]. Mechanistically, UFM1 suppresses gastric cancer invasion and metastasis by using ubiquitination to increase PDK1 degradation by negatively regulating PI3K/AKT signaling [109,110]. High UfBP1 expression increases the progression-free survival of advanced gastric cancer patients treated with platinum-based chemotherapy and enhances the sensitivity of gastric cancer cells to cisplatin. UfBP1 promotes the formation of K48-linked polyubiquitin chains on erythroid-2-related factor 2 (NRF2), a major antioxidant response element, and thus augments its proteasome-mediated degradation, which decreases the expression of aldo-keto reductase 1Cs (AKR1Cs) at the transcriptional level. In accordance with this finding, UfBP1 depletion significantly reduces the production of ROS in the presence of cisplatin. Therefore, UfBP1 enhances the sensitivity of gastric cancer cells to cisplatin via the NRF2/AKR1C axis. In osteosarcoma cells, UfBP1 depletion also attenuates NRF2 stability; however, it contributes to ROS accumulation, which promotes apoptosis and enhances chemosensitivity to doxorubicin and etoposide [113].

In renal cell carcinoma (RCC), UFL1 and UfBP1 expression are also downregulated and positively correlated with levels of p53 [21], a protein closely associated with multiple cancers. It is reported that p53 interacts with UFL1 and UfBP1 and is modified by UFM1 [21]. UFMylation mediated by UFL1 and UfBP1 stabilizes p53 by antagonizing MDM2-mediated ubiquitination and proteasome degradation, which inhibits cell growth and tumor formation *in vivo* [21]. Studies using mouse xenograft models in addition to a RCC tissue microarray analysis of 40 paired patient samples indicated that UFL1 and UfBP1 can act as tumor inhibitors by regulating p53 stability [21]. These results also demonstrated that UFMylation is a crucial PTM for the maintenance of p53 stability and tumor-suppressive function and implicated UFMylation as a promising therapeutic target in cancer. However, another study showed that UFMylation was significantly upregulated [114]; therefore, more research is needed to form a satisfactory explanation.

In breast cancer, targeting the estrogen receptor-a (ERα) is a promising strategy for prevention and therapy. ERα is a classical nuclear receptor for estrogen that can regulate the transcription of targeting genes [115] and is UFMylated at K171 and K180 [27]. UBA5 depletion causes decreased ERα stability, whereas UfSP2 depletion enhances ERα stability [27]. A UFMylation-defective mutation (2KR: K171R and K180R) markedly reduces ERα stability, confirming that ERα UFMylation inhibits the ubiquitin-proteasome-dependent degradation of the ERα [27]. Ectopic expression of ERα, but not the 2KR mutant, increased the mRNA levels of *ERα* target genes, such as those encoding pS2, cyclin D1, and c-Myc, suggesting that UFMylation promotes ERα transactivation [27]. In addition, overexpression of *ERα*, but not the 2KR mutant, promoted cell proliferation [27]. More importantly, all the UFMylation system factors, including UBA5, UFC1, UFL1, UFM1, and UfSP2, were highly expressed in ERα-positive breast cancer cell lines and tissues [27]. Collectively, these findings indicate a critical role for ERα UFMylation in breast cancer development by ameliorating the stability and transcriptional activity of the receptor. In addition, ASC1, a transcriptional coactivator of ERα, was also revealed to be UFMylated at K324/325/334/367, with UfBP1 playing an important role [17]. These modifications occurred only under conditions of 17β-estradiol treatment and could be reversed by the binding of UfSP2 to the zinc finger (ZnF) domain of ASC1 [17]. ERα competes with UfSP2 to bind to the ZnF domain of ASC1, thereby promoting ASC1 UFMylation. UFMylated ASC1 acts as a scaffold to enhance the association between p300, SRC1, itself and the promoters of ERα target genes, thereby promoting the proliferation of a large subset of breast tumor cells [17]. Moreover, UfSP2 depletion exacerbated ERα-mediated tumor formation; however, the expression of an ASC1-UFMylation-defective mutant or the depletion of UBA5 inhibited tumor growth, suggesting that the UFMylation of ASC1 is important for the transactivation of ERα and thus breast cancer development [17]. These results suggest that the UFMylation of ASC1 is vital for ERα transactivation and breast cancer progression.

SLC7A11, a key component of the cystine–glutamate antiporter [116], is another UFMylation substrate with important functions in breast cancers. In contrast to ACS1 and ERα UFMylation, SLC7A11 UFMylation inhibits metformin-induced breast cancer cell ferroptosis [29]. Metformin can induce ferroptosis in breast cancer cell lines and, therefore, suppress tumor growth in a manner independent of canonical AMPK signaling. UFMylation stabilizes SALC7A11 and metformin reduces SLC7A11 protein stability by inhibiting UFM1 expression, which induces ferroptosis to suppress cancer proliferation.

Recently, two other substrates of UFMylation, PLAC8 [31] and PD-L1 [31], were identified as playing significant roles in breast cancer pathogenesis. Mao et al. reported that PLAC8 was generally expressed at high levels in triple-negative breast cancer (TNBC) and UFMylated at K103 to maintain its protein stability [31]. Stabilized PLAC8 interacts with glycosylated and ubiquitinated PD-L1, which upregulates the levels of PD-L1, thereby promoting cancer cell proliferation and inhibiting the immune response [31]. PD-L1 can also be UFMylated at six lysines (K75/89/105/162/280/281). In contrast with PLAC8, UFMylation destabilizes PD-L1 by synergizing its ubiquitination [32]. Inhibiting PD-L1 UFMylation by silencing UFL1 or UFM1, or by the defective UFMylation of PD-L1, stabilized PD-L1 in multiple human and murine cancer cells and undermined antitumor immunity both in vitro and in mice [32].

In summary, evidence suggests that UFMylation has various roles in multiple cancers (Table 2) and indicates the potential of targeting the UFMylation cascade as a novel and promising strategy for tumor therapy.

## 4. Future Perspectives

Ubiquitin can be covalently linked to targeted substrates or ubiquitin molecules already attached to a substrate as monomers or polymers, resulting in mono- or polyubiquitination. Polyubiquitination chains can be formed using seven different lysines in ubiquitin (K6/11/27/29/33/48/63) or the starting methionine (M1) [3]. These ubiquitination patterns determine the fate of substrate proteins and activate different signaling pathways and biological functions. For instance, K48-linked ubiquitin chains regulate proteasomal degradation, and K63-linked chains regulate the DNA damage response and mitophagy [117]. Similar to Ub, UFM1 can also bind covalently to its substrate through a three-enzyme cascade to form mono- or poly-UFM1 chains. Although UFM1 contains six lysine residues, K69-linked UFM1 chains are the only type of poly-UFMylation reported so far [17]. Whether other lysine-mediated UFM1 chains exist is still unclear.

Although hundreds of E3 ligases function within the ubiquitination system, UFL1 is the only E3 ligase identified for UFMylation to date. Two UFL1-related proteins, UfBP1 and CDK5RAP3, which are necessary for UFL1 function and stability have been identified so far. Recently, data suggest that UFL1 has no specific active core and might function as a scaffolding protein, similar to RING-type E3 ligases [47]. With the aid of UfBP1 and CDK5RAP3, UFL1 brings UFC1 close to its substrates and promotes the transfer of UFM1. Considering the importance of E3s for ubiquitination and other UBLylations, other E3 ligases may be involved in UFMylation, and further research is needed to clarify this issue.

At present, there are two challenges facing UFMylation research: the identification of UFMylation substrates and sites, and the development of selective UFMylation inhibitors. Mass spectrometry (MS)-based proteomic analyses are widely used to identify ubiquitination and UBLylation substrates and modification sites [118,119]. MS is also commonly used for the identification of UFMylation substrates. However, commercial UFM1 antibodies have low affinity, sensitivity, and specificity, and therefore cannot be used effectively for the enrichment of UFMylation substrate groups. To date, almost all attempts at screening for UFMylation substrates have used exogenously expressed and tagged UFM1 to enrich UFMylation substrates selectively [23,24,77,78,118,119]. Unfortunately, the use of exogenous and redundant tags means that this method cannot adequately reflect the reality of endogenous UFMylation. In addition, the percentage of UFMylated peptides in the global proteome is extremely low, which further decreases the sensitivity of MS and increases the difficulty in identifying UFMylation substrates and modification sites. There are also no successful cases of the use of MS for identification of UFMylation sites with low modification rates. Therefore, a chemical tool to enrich UFMylated proteins is urgently required to identify UFMylation sites.

Recently, one group reported using an antibody-free approach for ubiquitination profiling (AFUP) that involved selectively clicking the ubiquitinated lysines of substrates to enrich and profile endogenous ubiquitinated peptides using MS [120]. AFUP includes four major steps: (1) blocking all free amino groups, including lysine ε-NH2 and protein N-terminal α-NH2, with formaldehyde at the protein level; (2) hydrolyzing ubiquitin chains using USP2 and USP21 to generate free lysine ε-NH2 at ubiquitination sites; (3) clicking the free lysine ε-NH2 using NHS-SS-biotin reagents, which specifically and sensitively react with the exposed lysine ε-NH2, followed by enrichment of labeled peptides using streptavidin sepharose beads; and (4) eluting and analyzing peptides by liquid chromatography-tandem mass spectrometry (LC-MS/MS). We propose that, with some modifications, AFUP is suitable for use in UFMylated peptide profiling. If the USP1 and USP21 in the second step were replaced with UfSP1 and UfSP2 to expose the lysine ε-NH2 of UFMylated proteins, this could be an excellent strategy to not only enrich UFMylated proteins, but also to identify the UFMylated sites of the substrates.

The development of selective inhibitors represents another challenge for those researching UFMylation. UFMylation is closely related to the occurrence and development of many cancers, suggesting that components of the UFMylation system are potential therapeutic targets in cancer. To date, several inhibitors of UFMylation have been discovered. Adenosine 5′-sulfamate, the mechanism-based pan-E1 inhibitor, binds tightly to the active site of UBA5 to prevent further substrate binding or catalysis [118]. Usenamine A, a natural product of the lichen *Usnea longissimi*, occupies the binding interfaces between UBA5 and UFM1 to inhibit UBA5 activity and attenuate the development and progression of breast cancer [105]. Compound-8, a covalent inhibitor of UfSP2, has also been found to promote UFMylation activity and contribute to immunotherapy with an antibody against PD-L1 [32]. Therefore, chemicals targeting UFMylation present novel and promising therapeutic strategies for cancers. The above chemicals are beginning to be developed to generate UFMylation inhibitors and tumor-targeting therapeutic drugs. However, the lack of chemical candidates and specificity has hindered the application of UFMylation targets in tumor therapy. 

## 5. Conclusions

Research on UFMylation over the past 20 years has yielded an understanding of the basic biological mechanism of UFMylation and the components of the UFMylation system; however, an in-depth understanding of UFMylation is still lacking. Accumulating evidence shows that UFMylation is closely related to the occurrence and development of human diseases. In this review, we have summarized the role and specific molecular mechanisms of UFMylation in five important cellular life activities, including the DNA damage response, ER metabolism, development, immune responses, and cancer. Whether UFMylation is involved in other cellular activities remains to be fully elucidated. Going forwards, UFMylation research must focus on clarifying whether additional UFMylation E3 ligases exist, developing effective strategies for UFM1 substrate screening, and identifying whether there are other UFM1 linking sites besides K69. Despite the hurdles that must be overcome, UFMylation is attracting increasing attention as a crucial PTM, and, in the clinical context, it is likely to provide the basis for new and effective cancer therapies.

## Figures and Tables

**Table 1:** A summary of verified UFMylation substrates.

UFMylation Substrate	Modification Sites	Function	Ref.
UfBP1	K267	Maintains ER homeostasis	[19]
UFC1	K122	UFMylation inhibition	[20]
ASC1	K324, K325, K334, and K367	Promotes ERα transactivation and breast cancer progression	[17]
p53	K351, K357, K370, and K373	Maintains p53 stability and suppresses tumor growth	[21]
RPN1	Unknown	Mediates ER-phagy	[22]
CYB5R3	K214	Mediates ER-phagy	[23]
RPL26	K132 and K134	Mediates ER-phagy	[24]
MRE11	K282	Promotes MRN complex formation and recruitment, ATM activation, DSB repair, and genome stability	[25]
Histone H4	K31	Amplifies ATM activation and maintains genomic integrity	[26]
ERα	K171 and K180	Increases ERα stability and transactivity and promotes breast cancer cell proliferation	[27]
RPL10	K30 and K101	Enhances pancreatic cancer cell stemness for PAAD development	[28]
SLC7A11	Unknown	Stabilizes SCL7A11 and inhibits metformin-induced breast cancer cell ferroptosis	[29]
14-3-3ε	Unknown	Promotes its interaction with RIG-I and activates MAVS, which induces IFN production and the antiviral innate immune response after RNA virus infection	[30]
PLAC8	K103	Promotes cancer cell proliferation and affects the immune response by regulating levels of PD-L1 ubiquitination	[31]
PD-L1	K75, K89, K105, K162, K280, and K281	Dysregulation of PD-L1 by UFMylation boosts tumor immune evasion	[32]
P4HB	K69, K114 and K130	UFMylation stabilizes P4HB, which inhibits oxidative and ER stresses and guarantees mitochondrial function	[33]

**Table 2:** The function of UFMylation in various cancers.

Cancer Type	Gene	Function	Ref.
Breast cancer	*ERα*	Promoting ERα stability and transactivity to promote breast cancer development	[27]
	*ASC1*	UFMylation of ASC1 promotes breast cancer cell growth and tumor formation	[17]
	*SLC7A11*	UFMylation stabilizes SALC7A11 and metformin reduces the protein stability of SLC7A11 by reducing UFM1	[29]
	*PLAC8*	UFMylation of PLAC8 may influence tumor progression and immune response in TNBC cells by reducing PD-L1 ubiquitination	[31]
	*PD-L1*	UFMylation of PD-L1 destabilizes PD-L1 by acting synergistically to promote its ubiquitination	[32]
Colon cancer	*UfSP2*	UfSP2 depletion significantly promotes the growth of tumor cells	[105]
	*UBA5*	Inhibition of UBA5 impedes tumor development	[106]
Gastric cancer	*UFM1*	UFM1 suppresses the invasion and metastasis of gastric cancer by increasing the degradation of PDK1 through ubiquitination mediated by negatively regulating PI3K/AKT signaling	[109,110]
	*UfBP1*	UfBP1 enhances the sensitivity of gastric cancer cells to cisplatin via the NRF2/AKR1C axis	[113]
	*CDK5RAP3*	CDK5RAP3 suppresses the development of gastric cancer by inhibiting the phosphorylation of AKT/GSK-3β and negatively regulating Wnt/β-catenin signaling	[111,112]
Osteosarcoma	*UfBP1*	UfBP1 depletion promotes apoptosis and enhances the chemosensitivity of cells to doxorubicin and etoposide	[113]
Renal cell carcinomas	*p53*	UFMylation stabilizes p53 by inhibiting its ubiquitination, which suppresses cell growth and tumor formation	[21]
Pancreatic adenocarcinoma	*RPL10*	PRL10 UFMylation enhances pancreatic cancer cell stemness for PAAD development	[28]
Hepatocellular carcinoma	*UFM1*	B3GALT5-AS1 regulates miR-934 and UFM1 to achieve negative regulation of HCC cell proliferation, invasion, and metastasis	[107]
	*UFL1* and *UfBP1*	UFL1 and UfBP1 act as gatekeepers to prevent liver fibrosis and subsequent steatohepatitis and HCC development by inhibiting the mTOR pathway	[96]
